# Three distinct outcomes in patients with colorectal adenocarcinoma and lymphovascular invasion: the good, the bad, and the ugly

**DOI:** 10.1007/s00384-021-04004-7

**Published:** 2021-08-21

**Authors:** Giorgio Bianchi, Alfredo Annicchiarico, Andrea Morini, Lorenzo Pagliai, Pellegrino Crafa, Francesco Leonardi, Paolo Dell’Abate, Renato Costi

**Affiliations:** 1 Dipartimento di Medicina e Chirurgia, Università di Parma, Parma, Italia; 2 Unità Operativa di Anatomia Patologica, Ospedale Maggiore di Parma, Azienda Ospedaliero-Universitaria di Parma, Parma, Italia; 3 Unità Operativa di Oncologia, Ospedale Maggiore di Parma, Azienda Ospedaliero-Universitaria di Parma, Parma, Italia; 4 Unità Operativa di Chirurgia Generale, Ospedale Maggiore di Parma, Azienda Ospedaliero-Universitaria di Parma, Parma, Italia; 5 Operativa di Chirurgia Generale, Sede ulteriore dell‛Università di Parma, Ospedale di Fidenza-Vaio, Azienda Sanitaria Locale (ASL) di Parma, Fidenza (Parma), Italia

**Keywords:** Colon, Rectum, Cancer, Lymphovascular invasion, Survival

## Abstract

**Purpose:**

In colorectal cancer (CRC), lymphovascular invasion (LVI) is a predictor of poor outcome and its analysis is nowadays recommended. Literature is still extremely heterogeneous, and we hypothesize that, within such a group of patients, there are any further predictors of survival.

**Methods:**

A total of 2652 patients with I–III-stage CRC undergoing resection between 2002 and 2018 were included in a retrospective analysis of demographic, clinical, and histology with the aim of defining the impact of LVI on overall survival (OS) and its relationship with other prognostic factors.

**Results:**

Overall, 5-year-OS was 62.6% (77-month-median survival). LVI was found in 558 (21%) specimens and resulted associated with 44.9%-5-year-OS (44 months) vs. 64.1% (104 months) of LVI cases. At multivariate analysis, LVI (*p* = 0.009), T3–4 (*p* < 0.001), and N ≠ 0 (*p* < 0.001) resulted independent predictors of outcome. LVI resulted as being associated with older age (*p* < 0.013), T3–4 (*p* < 0.001), lower grading (*p* < 0.001), N ≠ 0 (*p* < 0.001), mucinous histology (*p* < 0.001), budding (*p* < 0.001), and PNI (*p* < 0.001).

Within the LVI + patients, T3–4 (*p* = 0.009) and N ≠ 0 (*p* < 0.001) resulted as independent predictors of shortened OS. In particular, N-status impacted the prognosis of patients with T3–4 tumors (*p* = 0.020), whereas it did not impact the prognosis of patients with T1–2 tumors (*p* = 0.393). Three groups (T1–2anyN, T3–4N0, T3–4 N ≠ 0), with distinct outcome (approximately 70%-, 52%-, and 35%-5-year-OS, respectively), were identified.

**Conclusions:**

LVI is associated with more aggressive/more advanced CRC and is confirmed as predictor of poor outcome. By using T- and N-stage, a simple algorithm may easily allow re-assessing the expected survival of patients with LVI + tumors.

## Introduction

Colorectal cancer (CRC) is the third most frequently diagnosed neoplasia and the third cause of cancer-related death in the USA and worldwide [1, 2].

CRC long-term outcome has been associated to a variety of demographic, clinical, surgical, and histopathological criteria [3]. Lymphatic system is a major metastatic pathway of CRC and, since the sixties, lymph node status is used to assess tumor stage, patient prognosis, and treatment modality [4, 5]. In the late eighties [6], LVI, defined as presence of tumor cells in the lymphatic system and vascular structures, has been introduced and has progressively gained acceptance as predictor of poor outcome [3, 7, 8]. Since, LVI has been given full consideration as predictor of long-term survival of patients affected by CRC, and its analysis has been included in recommendations by international guidelines [3].

Although LVI is widely considered a major prognostic factor for CRC prognosis assessment, unfortunately, literature and recommendations on the subject are extremely heterogeneous and mostly based on large, heterogeneous registry-based surveys [9] or retrospective, small-sized series [10–13]. Moreover, it is still unclear whether, within the group of patients affected by CRC presenting LVI, there are further predictors of good or poor outcome.

In our large mono-centric retrospective survival analysis, the primary outcome was to verify and quantify the predictive role of LVI in stage I–III colorectal cancer undergoing curative surgery with respect of other demographic, clinical, surgical, and histopathological criteria. Secondary outcome was, within the group of LVI-positive patients, to identify criteria affecting the survival, in order to allow for a better assessment of long-term survival.

## Material and methods

### Patients and data acquisition

Demographic, clinical and pathological data, and oncological results of all consecutive patients undergoing curative surgery for histologically confirmed colon and rectal adenocarcinoma at the Department of Pathology at the Parma University Hospital were retrospectively reviewed.

Between January 2002 and December 2018, 2920 patients underwent surgery with curative purpose for colorectal adenocarcinoma. Before surgery, all patients with CRC diagnosed at preoperative endoscopy were staged according to the 8th edition of AJCC staging system [14]. Preoperative chest/abdominal/pelvic CT-scan and, for rectal cancer also pelvic MRI and/or endorectal ultrasound, were systematically performed for staging. Patients with extraperitoneal rectal cancer, < 80-years-old, and fit for neoadjuvant management underwent preoperative chemo-radiotherapy 6 to 8 weeks prior to surgery.

Patients less than 18-years-old, affected by Tis CRC as well as by synchronous metastatic disease (M ≠ 0, identified preoperatively or at surgery)/multiple cancers, undergoing non-radical or atypical colorectal resection (see list of recorded typical resections below) or eventually presenting less than 8 lymph nodes analyzed at pathology examination, were excluded from the study. A total of 2652 patients were included. Individuals who did not have follow-up survival data were not included in univariate/multivariate survival evaluations (steps 1 and 3). This study was reviewed and approved by the local Institutional Review Board.

### Management

Patient management was discussed at a preoperative multidisciplinary team meeting. Patients affected by rectal cancer underwent pelvic MRI and/or endorectal ultrasound, received standard NA-CRT (45 Gy in 25 fractions over a 5-week period with a combination of oxaliplatin and 5-fluorouracil), and, 6 to 8 weeks later, underwent curative surgical resection.

All patients underwent CRC curative surgical resection, namely right colectomy, splenic flexure segmental resection, left colectomy, rectal anterior resection, or abdominoperineal resection. *Right colectomy* procedure implied the ileo-colic and right colic vessel ligation at the origin, the complete mobilization of the hepatic flexure, and transverse colon resection ≥ 5 cm distally to the distal margin of the tumor; *splenic flexure segmental resection* was performed by left colonic flexure dissection until reaching the anterior aspect of the pancreas and left colic vessel ligation at the origin; *left colectomy* procedure included the inferior mesenteric vessel ligation at the origin, the complete mobilization of the splenic flexure, and colon resection at the recto-sigmoid junction; *rectal anterior resection* procedure implied the inferior mesenteric vessel ligation at the origin, the complete mobilization of the splenic flexure, total mesorectal excision, and rectum resection ≥ 1 cm distally to the distal margin of the tumor; *abdominoperineal resection* consisted in the dissection/ligature of the inferior mesenteric artery distally to the left colic artery origin, total mesorectum excision, internal/external anal sphincter amputation, and descending colon terminal colostomy in the left iliac fossa. Surgery was performed by laparotomy or laparoscopy depending on patient’s conditions, anesthesiologist’s evaluation, and surgeon’s preference.

Patients were offered adjuvant chemotherapy, tailored on an individual basis. Follow-up included physical examination, CEA serum level, colonoscopy, and CT/US every 6 months for the first 3 years, and annually thereafter, until the fifth postoperative year.

### Pathology and definition of lymphovascular invasion

Standard pathologic analysis was performed on formalin-fixed, paraffin-embedded tissue samples were cut into 4-μm sections and stained using hematoxylin and eosin (H&E) following radical colorectal resection specimens.

Tumor location was defined as the right colon (cecum, ascending colon, hepatic flexure, and transverse colon), left colon (descending and sigmoid colon), or rectum. Histology grade was classified according to the 8th UICC TNM staging system [14]. Resection specimens were evaluated for depth of tumor penetration (T), lymph node involvement (N), differentiation grade, mucinous component, existence of necrosis, signet-ring cell, tumor budding, perineural invasion (PNI), and LVI.

The assignment of the tumor grading was based on the number (percentage) of glandular formations found in the neoplasm. Accordingly, it was possible to define G1 — well-differentiated neoplasm, with gland formation > 95%, G2 — moderately differentiated neoplasm, with gland formation ranging between 50 and 95%, and G3 — poorly differentiated neoplasm, with gland formation ranging between 0 and > 49%. Undifferentiated carcinoma category (G4) was associated with no gland formation, squamous, or sarcomatoid differentiation. Tumor budding was defined by the presence of isolated single cancer cell or a cluster of fewer than 5 cancer cells were at the invasive front of the tumor [15]. PNI was defined according to Batsakis as tumor cell invasion in, around, and through the nerves in neurotropic carcinomas [16].

LVI was assessed according to the guidelines of the College of American Pathologists and was defined as presence of cancer cells within endothelial-lined channels, with the aid of immunohistochemical techniques (CD31) in doubtful cases. The distinction between lymphatic-invasion and blood vessel invasion was made sistematically [3].

### Study design and statistical analysis

The study was designed in three consecutive steps. The first step of the study was aimed to identify criteria associated with poor survival. During the second part of the study, the association between the presence of LVI and other demographic, clinical, and pathological factors was assessed in the whole population. The third analysis was performed within the group of LVI-positive (LVI +) patients and was aimed at identifying specific predictors of poor outcome; the results of this latter study were then used to create a reliable, easy-to-use classification system able to improve LVI + patients’ prognostic assessment and, at the same time, to enter clinical practice.

Quantitative variables were presented as mean. Categorical variables were presented as numbers and percentages. Comparisons of quantitative variables were performed using a Mann–Whitney test. Comparison of categorical variables was performed using Pearson’s chi-squared test, Fisher’s exact test, or the Mann–Whitney *U* test depending on numbers. Overall survival probabilities were calculated using the Kaplan–Meier method and survival curves were compared by using the log-rank test. Cox proportional hazards model was used for multivariate logistic regression analysis for factors with a *p* value of < 0.05 in univariate analysis. Data differences between groups were considered statistically significant at *p* < 0.05.

Analyses were performed using the SPSS software (version 11; SPSS, Inc, Chicago, IL).

## Results

A total amount of 2652 consecutive patients undergoing radical management of stage I–III CRC, including 558 presenting LVI at tumor specimen histology (21%), were eventually enrolled in the analysis. There were 1216 (45.9%) women and 1436 (54.1%) men, and mean age was 71.6 years (SD 11.6) overall. In 1252 (47.2%) patients, CRC was located in the right colon, 1041 (39.3%) in the left colon and 359 (13.5%) in the rectum. Complete follow-up data were available for 2237 patients (mean follow-up 54.2 months — SD 44.5), whose survival was studied for long-term analysis. Cumulative 5-year-overall survival (OS) rate was 62.6% (median survival 77 months — SE 1.2).

## Predictors of CRC long-term outcome

Univariate and multivariate (logistic regression) analyses of the association between several demographic, clinical, surgical, and pathological criteria are reported in Table 1.

**Table 1:** Clinicopathological factors and survival rates (No. 2652)

**Variable**		**Patients (No.)**	**%**	**Overall survival**			**Univariate analysis**	**Multivariate analysis**		
				**5-year survival rate**	**SE**	**Median (months)**	***p***	**OR**	**95%CI**	***p***
**Gender**										
	Male	1436	54.1	59.8	1.6	100	0.349			
	Female	1216	45.9	61.4	1.7	91				
**Location**										
	Colon	2293	86.5	61.8	1.2	98	0.150			
	Rectum	359	13.5	52.1	3.4	70				
**Location**										
	Right colon	1252	47.2	58.5	1.7	87	0.236			
	Left colon	1041	39.3	65.6	1.8	108				
	Rectum	359	13.5	52.7	3.4	70				
Stage (AJCC)*										
	Stage I	461	17.4	80.7	2.2	162	** < 0.001**			
	Stage II	982	37.0	67.6	1.8	115				
	Stage III	1038	39.1	45.6	1.9	47				
**Grading**										
	G1	310	11.7	66.4	3.4	99	** < 0.001**	1.056	0.945–1.180	0.341
	G2	1382	52.1	64.0	1.6	104				
	G3	872	32.9	52.8	2.0	68				
**Grading grouped**										
	G1–G2	1692	63.8	64.4	1.4	104	** < 0.001**			
	G3	872	32.9	52.8	2.0	68				
**pT**										
	pT1	111	4.2	86.8	3.7	140	** < 0.001**	1.51	1.348–1.690	** < 0.001**
	pT2	459	17.3	76.2	2.4	120				
	pT3	1158	58.7	60.2	1.5	92				
	pT4	373	14.1	38.5	3.0	31				
**T grouped**										
	T1; T2	570	21.5	78.2	2.1	150	** < 0.001**			
	T3; T4	1931	72.8	55.9	1.4	81				
**Node status**										
	N0	1445	54.5	72.1	1.4	130	** < 0.001**			
	N1	611	23.0	53.3	2.5	64				
	N2	424	16.0	34.5	2.8	28				
**Node status**										
	N = 0	1445	54.5	72.1	1.4	130	** < 0.001**	1.48	1.358–1.620	** < 0.001**
	N ≠ 0	1035	39.0	45.5	1.9	47				
**LVI**										
	No	2094	79.0	64.1	1.2	104	** < 0.001**	1.301	1.068–1.584	**0.009**
	Yes	558	21.0	44.9	3.0	44				
**PNI**										
	No	2533	95.5	61.6	1.2	97	** < 0.001**	1.28	0.917–1.784	0.147
	Yes	115	4.3	35.2	6.5	26				
**Mucinous**										
	No	1456	92.6	61.2	1.2	97	**0.004**	1.189	0.905–1.562	0.214
	Yes	196	7.4	53.3	4.9	61				
**Signet-ring cells**										
	No	2632	99.2	60.7	1.2	95	0.381			
	Yes	20	0.8	57.3	12.7	68				
**Ulcerated**										
	No	2581	97.3	60.8	1.2	96	0.185			
	Yes	71	2.7	56.0	6.8	82				
**Budding**										
	No	2331	87.9	61.8	1.2	98	** < 0.001**	1.161	0.901–1.497	0.247
	Yes	321	12.1	47.6	5.1	56				
**Lymph nodes**										
	< 12	179	10.1	63.6	4.0	94	0.961			
	≥ 12	1459	89.9	61.5	1.4	97				

*SE* standard error, *OR* odds ratio *CI* confidence interval, *LVI*, lymphovascular invasion, *PNI* perineural invasion

*According to the 8th edition of AJCC staging system^14^

At univariate analysis, factors associated with poorer overall survival were grading (*p* < 0.001), T (*p* < 0.001), N (*p* < 0.001), LVI (*p* < 0.001), PNI (*p* < 0.001), mucinous histology (mucinous component exceeding 50%) (*p* = 0.004), and budding (*p* < 0.001).

**Fig. 1:**
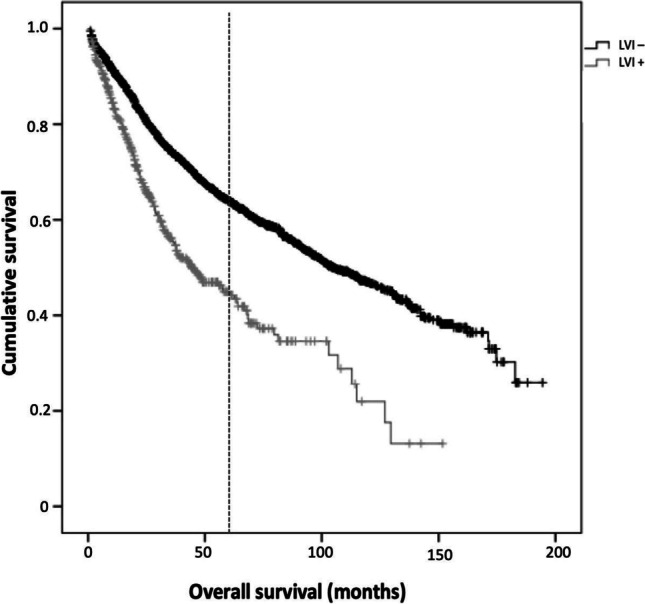
Overall survival according to the presence of lymphovascular invasion. Legend: LVI, lymphovascular invasion

At multivariate analysis, LVI was confirmed as independent prognostic factor (OR 1.301; 95%CI 1.06–1.58; *p* = 0.009) as well as T (OR 1.510; 95%CI 1.34–1.69; *p* < 0.001 and N (OR 1.483; 95%CI 1.35–1.62; *p* < 0.001). Five-year-survival rate of LVI + patients was significantly lower (*p* < 0.001) compared with that of LVI-negative (LVI −) tumors, resulting as being 44.9% (SE 3.0; median survival 44 months) vs. 64.1% (SE 1.2; median survival 104 months) (Fig. 1).

## Criteria associated with LVI

LVI resulted associated with higher depth of invasion (*p* < 0.001), lower grading (*p* < 0.001), presence of lymph node metastasis (*p* < 0.001), mucinous histology (*p* < 0.001), tumor budding (*p* < 0.001), and PNI (*p* < 0.001) (Table 2). Among tumors with lymph node metastasis, those presenting LVI were also associated with a higher number of metastatic nodes (2.76 SD 5.34 vs. 1.25 SD 2.93; *p* < 0.001).

**Table 2:** Correlation between LVI and clinicopathological factors of colorectal cancer patients

		**LVI**				***p***
		**Negative (*n* = 2094)**	**% (SD)**	**Positive (*n* = 558)**	**% (SD)**	
**Age (years)**						
	< 60	353	77.20	104	22.80	0.344
	> 60	1741	79.30	454	20.70	
**Gender**						
	Male	1142	78.30	294	21.70	0.445
	Female	952	79.50	264	20.50	
**Location**						
	Right colon	987	78.83	265	21.17	0.757
	Left colon	828	79.54	213	20.46	
	Rectum	279	77.72	80	22.28	
**pT**						
	pT1	102	91.89	9	8.11	** < 0.001**
	pT2	421	91.72	38	8.28	
	pT3	1211	77.73	347	22.27	
	pT4	224	60.05	149	39.95	
**Node status**						
	N0	1250	86.51	195	13.49	** < 0.001**
	N1	418	68.41	193	31.59	
	N2	273	64.39	151	35.61	
**Node status**						
	N = 0	1250	86.51	195	13.49	** < 0.001**
	N ≠ 0	691	66.76	344	33.24	
Stage (AJCC)*						
	Stage I	433	93.93	28	6.07	** < 0.001**
	Stage II	815	82.99	167	17.01	
	Stage III	693	66.76	345	33.24	
**Mucinous**						
	Yes	132	67.35	64	32.65	** < 0.001**
	No	1962	79.89	494	20.11	
**Signet-ring cells**						
	Yes	16	80.00	4	20.00	0.987
	No	2078	78.95	554	21.05	
**Necrosis**						
	Yes	38	71.70	15	28.30	0.231
	No	2056	79.11	543	20.89	
**Grading**						
	G1	253	81.61	57	18.39	** < 0.001**
	G2	1155	83.57	227	16.43	
	G3	604	69.27	268	30.73	
**Budding**						
	Yes	96	29.91	225	70.09	** < 0.001**
	No	1998	85.71	333	14.29	
**PNI**						
	Positive	16	13.91	99	86.09	** < 0.001**
	Negative	2074	81.88	459	18.12	
**Lymph nodes (mean)**		20.44	10.11	21.09	12.54	0.097
**Metastatic nodes (mean)**		1.25	2.93	2.76	5.34	** < 0.001**

*SD* standard deviation, *LVI* lymphovascular invasion, *PNI* perineural invasion

*According to the 8th edition of AJCC staging system^14^

## Predictors of long-term outcome within the group of LVI + CRC patients

Concerning the subgroup of 558 patients affected by LVI + tumors, at univariate analysis, tumor differentiation (*p* = 0.004), depth of tumor invasion (*p* < 0.001), and lymph node metastasis (*p* < 0.001) were prognostic factors influencing overall survival. Multivariate analysis showed T (OR 1.627; 95%CI 1.27–2.00; *p* = 0.009) and N (OR 1.468; 95%CI 1.22–1.75; *p* < 0.001) as prognostic factors independently associated with shortened survival (Table 3).

**Table 3:** Clinicopathological factors and survival in LVI patients (No. 558)

**Variable**		**Patients (No.)**	**%**	**Overall Survival**			**Univariate analysis**	**Multivariate analysis**		
				**5-year-OS**	**SE**	**Median (months)**	***p***	**OR**	**95%CI**	***p***
**Gender**										
	Male	294	52.7	40.6	4.1	42.0	0.842			
	Female	264	47.3	45.5	3.9	39.4				
**Location**										
	Right colon	265	47.5	42.9	4.0	39.4	0.878			
	Left colon	213	38.2	48.2	4.8	48.7				
	Rectum	80	14.3	27.3	7.0	28.1				
Stage (AJCC)*										
	Stage I	28	5.2	67.1	10.5	89.1	** < 0.001**			
	Stage II	167	30.9	52.6	5.8	71.2				
	Stage III	345	63.9	36.9	3.5	48.8				
**Grading**										
	G1	57	10.3	61.8	9.0	68.3	**0.004**	1.188	0.954–1.479	0.125
	G2	227	41.1	46.1	4.6	42.0				
	G3	268	48.6	36.4	4.0	34.0				
**pT**										
	pT1	9	1.7	60.0	18.2	39.4	** < 0.001**	1.627	1.274–2.007	** < 0.001**
	pT2	38	7.0	71.9	9.3	98.0				
	pT3	347	63.9	47.3	3.8	63.2				
	pT4	149	27.4	25.1	4.6	36.3				
**Node status**										
	N0	195	36.2	55.0	5.2	67.0	** < 0.001**	1.468	1.226–1.758	** < 0.001**
	N1	193	35.8	44.6	5.2	46.9				
	N2	151	28.0	26.9	4.7	19.4				
	N ≠ 0	344	63,8	36,7	3,5	31,6				
**PNI**										
	No	459	82.3	43.2	3.1	43.6	0.376			
	Yes	99	17.7	42.0	6.9	26.9				
**Mucinous**										
	No	494	88.5	42.3	3.0	39.2	0.927			
	Yes	64	11.5	48.4	7.9	39.4				
**Signet-ring cells**										
	No	554	99.3	42.9	2.9	39.4	0.656			
	Yes	4	0.7	37.5	28.6	14.5				
**Ulcerated**										
	No	551	98.7	43.2	2.9	39.2	0.615			
	Yes	7	1.3	/		15.3				
**Budding**										
	No	333	59.7	43.0	3.3	43.6	0.865			
	Yes	225	40.3	44.5	5.1	37.7				
**Lymph nodes**										
	< 12	23	6.9	68.6	12.5	98.0	0.083			
	≥ 12	310	93.1	44.1	3.9	52.0				

*SE* standard error, *OR* odds ratio, *LVI* lymphovascular invasion, *PNI* perineural invasion

According to the 8th edition of AJCC staging system^14^

**Fig. 2:**
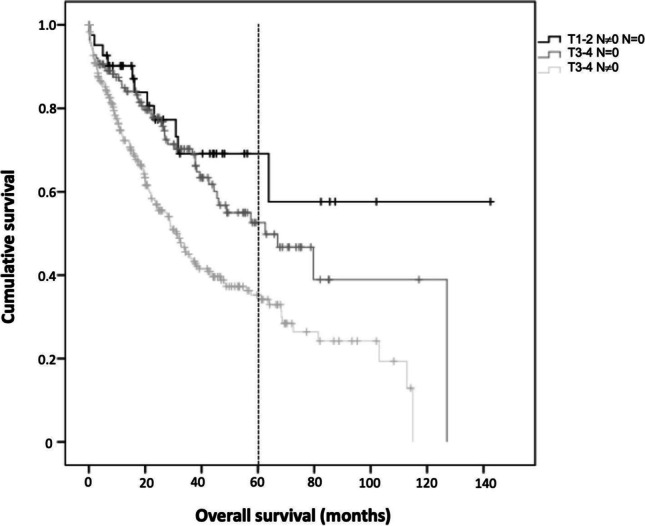
Overall survival in LVI + patients according to the T- and N-stage. LVI, lymphovascular invasion, N = 0, lymph nodes not involved, N ≠ 0, lymph node metastasis

In particular, as reported in Table 4, the presence of lymph node metastasis (N ≠ 0) resulted as being associated with a shortened survival in the case of patients affected by locally advanced (T3–4) tumors (*p* < 0.001), whereas lymph node status did not affect the prognosis of T1–2 tumors (*p* = 0.393). Thus, three groups of patients with distinct long-term prognosis are identified (Table 5, Fig. 2).

**Table 4:** LVI + patients’ outcome according to their T stage (T1–2 vs. T3–4) and N status (N ≠ 0 vs. N = 0)

Variable	Univariate analysis (OS)						
	**Nodal involvement**						
	**Negative (N = 0)**			**Positive (N ≠ 0)**			***p***
	**No**	**5 years, %**	**SE**	**No**	**5 years, %**	**SE**	
**T1–2**	26	67.1	10.5	18	70.7	14.6	0.393
**T3–4**	149	52.6	5.8	293	35.2	3.5	** < 0.001**

*LVI*, lymphovascular invasion, *OS* overall survival, *SE*, standard error, *N* = *0* lymph nodes not involved, *N ≠ 0* lymph node metastasis

**Table 5:** Three prognostic classes of LVI-positive CRC according to their T stage (T1–2 vs. T3–4) and N status (N ≠ 0 vs. N = 0)

T stage	N status	5-year-overall survival	Prognostic class
**T1–2**	Any N	67.1% (N = 0)/70.7% (N ≠ 0) (~ 2/3)	Class 1 (“Good”)
**T3–4**	N = 0	52% (~ 1/2)	Class 2 (“Bad”)
	N ≠ 0	35% (~ 1/3)	Class 3 (“Ugly”)

*LVI* lymphovascular invasion, *CRC* colorectal cancer, *N* = *0* lymph nodes not involved, *N ≠ 0* lymph node metastasis

## Discussion

Our study, analyzing the long-term outcome of 2692 patients through almost two decades, confirms LVI as a major predictor of poor prognosis after CRC radical resection, and, within LVI + population, eventually allows identifying three groups of patients with distinct expected survival.

With a 44.9% 5-year-OS (versus 64.1% of general population — Table 1), LVI resulted as being a strong predictor of poor outcome, significantly affecting CRC patients’ overall survival (*p* = 0.011; HR 1.28; 95%CI 1.06–1.56). In 1999, a consensus statement of the College of the American Pathologists and AJCC stratified prognostic factors in four categories reflecting the strength of their prognostic value, listing LVI among first category prognostic factor negatively affecting patients’ survival, together with local tumor extent (T), nodal involvement (N), distant metastasis (M), tumor budding, and residual tumor after surgical resection [3]. Nowadays LVI is widely accepted as strong negative prognostic factor and is listed by NCCN guidelines among the high-risk features for colon and rectal cancer, together with positive margins, bowel obstruction, < 12 lymph nodes examined, perineural invasion, localized perforation, and poorly differentiated histology [17].

As expected, in our cohort of patients, we also found that lymph node status was independently associated with a poor prognosis (*p* < 0.001). Such a finding was expected, as lymph nodes have traditionally been considered the first organs where carcinomas (in general) can develop metastasis [18], and, after several decades, lymph node metastasis is still considered a key step of CRC progression towards systemic spread and a pivotal issue in patient management and prognosis assessment [19].

LVI resulted as being associated with several other criteria (Table 2) including advanced T and N stage tumors, high grade, tumor budding, PNI, and mucinous histology. Among patients with nodal involvement, LVI + tumors had a higher number of metastatic lymph nodes (2.76 vs. 1.25), which roughly means that, in most cases, patients with LVI + and LVI-tumors present different TNM stages at pathology (N1b vs. N1a). Such a relationship of LVI and N status, at some level, confirms the hypothesis suggesting that LVI may be considered to be a precursor of, and therefore associated with, lymph node metastasis [20, 21], including occult ones [21, 22], eventually defining LVI as a potential predictor of patient outcome [23]. Significantly, LVI is found to be an independent risk factor of lymph node metastasis even in patients with T1 colorectal adenocarcinoma [24, 25].

As already showed by Lim et al., in our cohort of patients, LVI + tumors were more likely to present the following criteria: advanced T and N stage and high grade [7], but our results also showed an association between LVI + and three other criteria, namely tumor budding, PNI, and mucinous histology. Recently, tumor budding has gained interest and together with LVI and PNI represent as an independent prognostic factor in CRC and across a variety of other solid cancers [25–27]. As suggested by Kim et al. LVI, PNI, and tumor budding represent strong prognostic factors for stage I CRC and positive patients should receive close follow-up and potentially be considered for chemotherapy [25]. In our analysis, although tumor budding and PNI were related to worse survival at univariate analysis (Table 1) and were statistically associated to LVI (Table 2) surprisingly, neither was confirmed to be an independent prognostic factor at multivariate analysis both on the whole population examined and on LVI + only. This could be due to the scarcity of the patients examined; in fact, among LVI + patients (*n*. 558) those with PNI + and budding were respectively 99 and 225 and such a small number may not have reached the statistical power to confirm any association at the multivariate analysis.

After having confirmed LVI as an independent predictor of poor outcome (and associated with more aggressive tumors), further analysis was made among the 558 LVI + patients to explore which criteria eventually impacted on those patients’ already dismal prognosis. Such an analysis was aimed to identify patients with very short outcome and, conversely, others with not so poor outcome, in order to potentially tailor a more appropriate, specific management/follow-up. Interestingly, only advanced T (T3–4) and positive N status (N ≠ 0) were found to be independent prognostic factors at multivariate analysis (Table 3). Such a result, which was at some level non-expected, seemingly shows that, within this group of patients (accounting for roughly 1/5 of the total population), LVI annihilates the impact of most criteria traditionally associated with poor outcome.

Considering the two criteria independently associated with prognosis (T- and N-stage) within the group of LVI + patients, we eventually compared the outcome of four subgroups of patients pairing their T stage (T1–2 and T3–4) and N status (N ≠ 0 and N = 0). As expected, the majority of LVI + cases presented the criteria associated with shortened survival, namely advanced T (496, 91.3%) and N-stage (344, 63.8%). Despite in a recent meta-analysis Yuan et al. indicate that LVI is a negative prognostic factor also in patients with stage I-II CRC (T1-T4; N0) [28], in our series, surprisingly T-stage resulted as being the single most important predictor of outcome in patients with LVI + tumors, as an early T-stage (T1–2) resulted as being associated with better prognosis, regardless of N-status (Table 3). The 5-year-OS of these patients is in fact around 70% regardless of the state of lymph node invasion (N0 = 67.1%; N1 = 70.7%) and this places them in a risk class that is somewhat similar to the population with LVI- CRC (Table 5). Conversely, considering only patients with advanced T-stage (T3–4), the presence of lymph node metastases resulted as being associated with poorer outcome when compared to N0 cases (Table 4) and this defines other two prognostic classes (Table 5). In the field of hypotheses, in early-stage (T1–2) tumors, LVI may be associated to occult, micro-lymph node metastasis, as suggested by literature [21, 22], which may be supposed to have gone undetected in our study. Such an intriguing hypothesis could not be confirmed or refuted by our analysis, since pathology examination was performed in a traditional fashion. Alternatively, it may be hypothesized that, at an early T stage, the presence of LVI has a similar (if not the same) impact on outcome than lymph node metastasis, and therefore may not been considered just a precursor or a “preparatory” condition towards lymph node spread, but rather the early sign of a highly aggressive tumor behavior. Moreover, it may be supposed, at least hypothetically, that the immune response at lymph node station can enclose single tumor cells or small clusters and eliminate them in tumor’s early stages. In this light, the use of immunomodulators in the management of this group of patients could be of some interest. Unfortunately, patients with and a “reassessed” improved survival (being affected by LVI + , T1–2N_any_ tumor) are finally a small part of LVI + patients (44/558, 8%). On the other side, if we consider the prognosis associated with LVI + , T3–4N0 cases, and we admit that lymph node histology analysis may be suboptimal, those patients may probably be considered for the same management of N ≠ 0 patients, and therefore undergo adjuvant chemotherapy.

As a final result of our analyses, three prognostic classes of LVI + CRC could be identified:

1. patients with T1–2N_any_ tumors and, regardless of node status (N ≠ 0 and N = 0), a fairly “Good” prognosis, even comparable with LVI tumors (roughly 2/3 are alive 5 years postoperatively);
2. patients with locally advanced tumors and negative nodes (T3–4N0), presenting a “Bad” outcome (1/2 — or 52% — alive at 5 years);
3. patients with both advanced T-stage and positive nodes (T3–4 N ≠ 0), associated with an “Ugly” destiny (grossly 1/3 — 35% — alive 5 years postoperatively) (Table 4, Fig. 2).

Although our study was limited by relatively small numbers, retrospective nature of data collected through a long period of time, it should be remarked that the series was homogeneous, as patients were treated by the same team of surgeons/oncologists, and histology examined by the same team of pathologists, with a specific expertise in gastrointestinal tumors. This is the first study pondering the impact of prognostic factors within LVI+ patients, indeed; thus, unfortunately, it is impossible to compare our results with others. Nevertheless, since it mostly relies on traditional histopathology reports, our analysis is easily reproducible and may be possibly considered a preliminary step for larger scale studies.

## Conclusion

As expected, LVI resulted as being a major predictor of poor outcome and seems associated with more aggressive and/or more advanced CRC. Within the group of patients with LVI + tumors, T-stage is seemingly the most important predictor of outcome. Expected survival of LVI + cases may be easily and effectively reassessed according to a three-stage-classification based on T- and N-stage.
